# Development of a Novel Web-Based Tool to Enhance Clinical Skills in Medical Education

**DOI:** 10.2196/47438

**Published:** 2024-06-20

**Authors:** Ayma Aqib, Faiha Fareez, Elnaz Assadpour, Tubba Babar, Andrew Kokavec, Edward Wang, Thomas Lo, Jean-Paul Lam, Christopher Smith

**Affiliations:** 1 Schulich School of Medicine and Dentistry, Western University, London, ON, Canada; 2 Michael G. DeGroote School of Medicine, McMaster University, Hamilton, ON, Canada; 3 Department of Otolaryngology - Head and Neck Surgery, Schulich School of Medicine and Dentistry, Western University, London, ON, Canada; 4 GoodLabs Studio, Toronto, ON, Canada; 5 Department of Economics, University of Waterloo, Waterloo, ON, Canada

**Keywords:** medical education, objective structured clinical examination, OSCE, e-OSCE, Medical Council of Canada, MCC, virtual health, exam, examination, utility, usability, online learning, e-learning, medical student, medical students, clinical practice, clinical skills, clinical skill, OSCE tool

## Abstract

A significant component of Canadian medical education is the development of clinical skills. The medical educational curriculum assesses these skills through an objective structured clinical examination (OSCE). This OSCE assesses skills imperative to good clinical practice, such as patient communication, clinical decision-making, and medical knowledge. Despite the widespread implementation of this examination across all academic settings, few preparatory resources exist that cater specifically to Canadian medical students. MonkeyJacket is a novel, open-access, web-based application, built with the goal of providing medical students with an accessible and representative tool for clinical skill development for the OSCE and clinical settings. This viewpoint paper presents the development of the MonkeyJacket application and its potential to assist medical students in preparation for clinical examinations and practical settings. Limited resources exist that are web-based; accessible in terms of cost; specific to the Medical Council of Canada (MCC); and, most importantly, scalable in nature. The goal of this research study was to thoroughly describe the potential utility of the application, particularly its capacity to provide practice and scalable formative feedback to medical students. MonkeyJacket was developed to provide Canadian medical students with the opportunity to practice their clinical examination skills and receive peer feedback by using a centralized platform. The OSCE cases included in the application were developed by using the MCC guidelines to ensure their applicability to a Canadian setting. There are currently 75 cases covering 5 specialties, including cardiology, respirology, gastroenterology, neurology, and psychiatry. The MonkeyJacket application is a web-based platform that allows medical students to practice clinical decision-making skills in real time with their peers through a synchronous platform. Through this application, students can practice patient interviewing, clinical reasoning, developing differential diagnoses, and formulating a management plan, and they can receive both qualitative feedback and quantitative feedback. Each clinical case is associated with an assessment checklist that is accessible to students after practice sessions are complete; the checklist promotes personal improvement through peer feedback. This tool provides students with relevant case stems, follow-up questions that probe for differential diagnoses and management plans, assessment checklists, and the ability to review the trend in their performance. The MonkeyJacket application provides medical students with a valuable tool that promotes clinical skill development for OSCEs and clinical settings. MonkeyJacket introduces a way for medical learners to receive feedback regarding patient interviewing and clinical reasoning skills that is both formative and scalable in nature, in addition to promoting interinstitutional learning. The widespread use of this application can increase the practice of and feedback on clinical skills among medical learners. This will not only benefit the learner; more importantly, it can provide downstream benefits for the most valuable stakeholder in medicine—the patient.

## Introduction

In 2020 and 2021, over 5000 final-year medical students graduated from a Canadian medical program and were matched to a residency program [1]. For these cohorts, portions of in-person clinical learning were limited due to the COVID-19 pandemic. Alongside clinical learning, the COVID-19 pandemic also caused numerous academic and health care institutions to adopt more web-based learning platforms [2], thus emphasizing the importance of remote learning in the current day.

Prior to 2021, final-year Canadian medical students were required to pass an objective structured clinical examination (OSCE) held by the Medical Council of Canada (MCC) in order to progress to a residency training program [3]. Although this requirement has ceased for Canadian medical graduates, OSCEs remain integral within the medical education curriculum by serving as assessment tools for clinical skills. The goal of these OSCEs is to assess the candidate’s clinical judgment, reasoning, knowledge, and skills. The examination is typically divided into twelve 11-minute–long stations, with a 2-minute break between each station. Stations can include clinical problems within the following fields: internal medicine, surgery, pediatrics, obstetrics and gynecology, psychiatry, and preventative medicine and public health [3].

The resources available to medical students for OSCE preparation and the real-world clinical setting are few and far between. Although such resources exist, they are limited by one or more factors. One of the biggest limitations for existing OSCE resources is that they are not specific to the MCC objectives, thus restricting their use in a Canadian medical education setting. Another major limitation is that they are often not directed at medical students but rather at students in other health care disciplines, such as pharmacy students and nursing students. Although these resources are beneficial for practice purposes, other professions have different scopes of practice, and the OSCE feedback generated for students via such resources may not always be translatable. Additionally, many of the existing OSCE preparation tools require user setup with platforms such as Zoom or Microsoft Teams; there are few that exist as stand-alone applications through which students can access feedback, clinical prompts, and OSCE assessments within a single centralized platform.

Another important limitation of existing resources is the inability to provide users with feedback regarding their clinical performance, specifically through formative learning experiences. Clinical educators often utilize quantitative scores and feedback in the form of checklists in order to provide students with assessments of their performance. However, this may not always be possible, given the time constraints of clinicians and staff. A possible solution to this is the utilization of peer feedback through formative learning experiences [4]. Unlike summative assessments and examinations, formative learning experiences provide students with opportunities in which they are able to focus on skill development as opposed to percentages and grades. Several studies have demonstrated the benefits of formative experiences, such as encouraging reflective review, reducing test anxiety, and advancing the learners’ self-regulation skills [5,6]. Moreover, the remote nature of web-based platforms for formative learning can contribute to interinstructional learning, in which peers who have additional knowledge or exposure within certain medical fields can enhance the clinical skills of those whose training lacks in these areas.

Given the emphasis on web-based learning and the fact that few formative learning experiences exist for students, it is evident that there is a need for an electronic OSCE (e-OSCE) preparation tool that fills the aforementioned gaps in the medical education system. Thus, the beta version of the MonkeyJacket application for OSCE practice was developed with these gaps in mind [7]. The e-OSCE tool was piloted among a group of 6 medical students and resident physicians at Western University and McMaster University, with the goal of providing direct feedback to the software development team to refine the utility of the application. The primary research objective of this study was to describe the approach to the development and dissemination of the MonkeyJacket e-OSCE application tool. This paper also aims to describe the platform itself, the potential utility of the application as a tool that provides scalable formative feedback for learners, and how the application serves as a valuable tool in Canadian undergraduate medical education.

## Development

### Purpose of Development

The MonkeyJacket platform was built for the purpose of developing a formative learning experience (ie, rather than a summative one) in which the goals are to practice with various clinical cases and receive feedback through peer evaluations.

### Tool Development

The backend of the MonkeyJacket platform was developed by a team of software engineers, project managers, and data scientists. The platform, including the video chat functionality, was custom coded by using a combination of Jitsi (8x8 Inc) and JavaScript Node.js (OpenJS Foundation). Through numerous rounds of user testing and quality control, the application was consistently reviewed and improved by the development team to ensure a smooth experience for users.

### Development and Testing of the Application

The cases for the MonkeyJacket application were created by medical students and resident physicians. The trialing and testing of the application were conducted by a group of 6 medical students and resident physicians over a span of 3 months. Group members were encouraged to practice with everyone in the group to allow for diversity in perspectives and promote intragroup learning during the testing period. In addition to seeking group feedback regarding the practice cases and feedback checklists, the user study group was encouraged to provide feedback regarding functionality and ease of use. Comments were then relayed to the development team, and appropriate changes to the application were made.

### Inclusion of Cases

The goal was to build practice cases that address CanMED (communicator, collaborator, leader, health advocate, scholar, professional, and medical expert) roles and provide formative feedback in the following disciplines: cardiology, respirology, gastroenterology, neurology, and psychiatry [8-10]. Within each discipline, cases were developed based on common and vital red-flag clinical presentations across patient demographics. Additionally, some uncommon and highly fatal conditions were also included within the data set to represent the diversity of cases seen in clinical settings. There are a total of 75 cases in the data set, with no repeated diagnoses. All aspects of OSCEs, except the physical examination, were assessed. The cases were based on a composite of patient cases, of which some were created based on real-life deidentified scenarios, and others were adapted from an existing repertoire of cases from resources geared toward medical students, such as *OSCE and Clinical Skills Handbook* and other web-based resources [11-13]. Table 1 presents the number of cases per discipline.

**Table 1.** Breakdown of cases within the data set by medical discipline.

Medical discipline	Cases, n
Cardiology	14
Respirology	15
Gastroenterology	16
Neurology	15
Psychiatry	15

### Building the Physician Candidate Prompts

The next step was developing the clinical prompt and task for each case, for both the student presenting as the “patient” and the student practicing as the “physician.” We followed the MCC guidelines in ensuring that prompts were written in a clear and unambiguous manner and tasks could be completed in real time. For example, we avoided prompts such as “explore this further with the patient” and instead replaced them with prompts such as “take a thorough history, with a focus on GI symptoms and summarize your findings.” We also avoided time-defining phrases, such as “the symptoms started at 9am,” and instead replaced them with more definite timelines, such as “2 hours ago.” All clinical stems included the patient’s name, age, gender, and presenting symptoms and the task(s) that must be completed by the physician. The cases were framed such that it was the candidate’s first time assessing the patient, rather than assuming that they had a pre-existing relationship with the patient.

### Compiling Information for the Standardized Patient and Trainers

All patient case stems included the following demographic data: the patient’s name; age; occupation; opening statement or history of the presenting illness, including symptoms with qualifications (onset, duration, quality, severity, timeline, alleviating factors, etc); associated symptoms; past medical history; medication history; family history; and social history. For the latter items, only positive histories (eg, if the patient has a history of past illnesses or a family history) were given. Nonverbal cues were also indicated on the patient’s prompt so that they could be communicated to the physician, especially in psychiatry stations (eg, “I avoid eye contact, either looking at the ground or focusing on my hands. I give limited information making it obvious that I’m holding something back.”).

### Developing the Feedback Checklists

In deciding the number of checklist items for each clinical prompt, we included items that were relevant to assessing the candidate’s abilities and ensured that the checklists were not exhaustive. The number of items on each checklist depended on the complexity of the case, but most checklists consisted of 30 to 40 items. The checklist items all began with an action verb to guide the standardized patient, who is also the examiner, on what was expected from the physician.

Using the MCC guidelines, we ensured that the items were discrete, observable, and dichotomous. Toward ensuring that items were discrete, each checklist item assessed for 1 concept or grouped concepts together; the candidate could get the full score even if they asked about 1 concept within the group. For example, a checklist item for qualifying pain was “Elicits character of pain – sharp, dull.” For this checklist item, the candidate would get full marks for asking about any character of pain. In ensuring that items were observable, we avoided terminology including “understands” and “appreciates” and instead used terms like “asks about” and “gives reasonable differential diagnoses.” Toward ensuring that items were dichotomous, the candidate either received the full mark for the item or did not; the checklist did not have any rating scales or instructions regarding part marks.

### Review, Revise, and Pilot

The MCC states that case development is an iterative process requiring thought, review, and revision, and thus one should be open to feedback. The first step of the review involved the medical development team, which consisted of medical students and resident doctors, piloting the application in an iterative process to continue to refine the platform. This allowed us to identify missing information from the patient script and review the checklist to reduce ambiguity. Additionally, the cases were also reviewed by attending physicians in order to increase the validity of the clinical situations.

### Ethical Considerations

This study did not contain or capture any human information or data. Therefore, as per Article 2.4 from the Tri-Council Policy Statement Research Ethics Board, this study was exempt from research and ethics review and did not require research ethics board approval [14].

## Application Interface and Features

### Description of the Application

**Figure 1.**
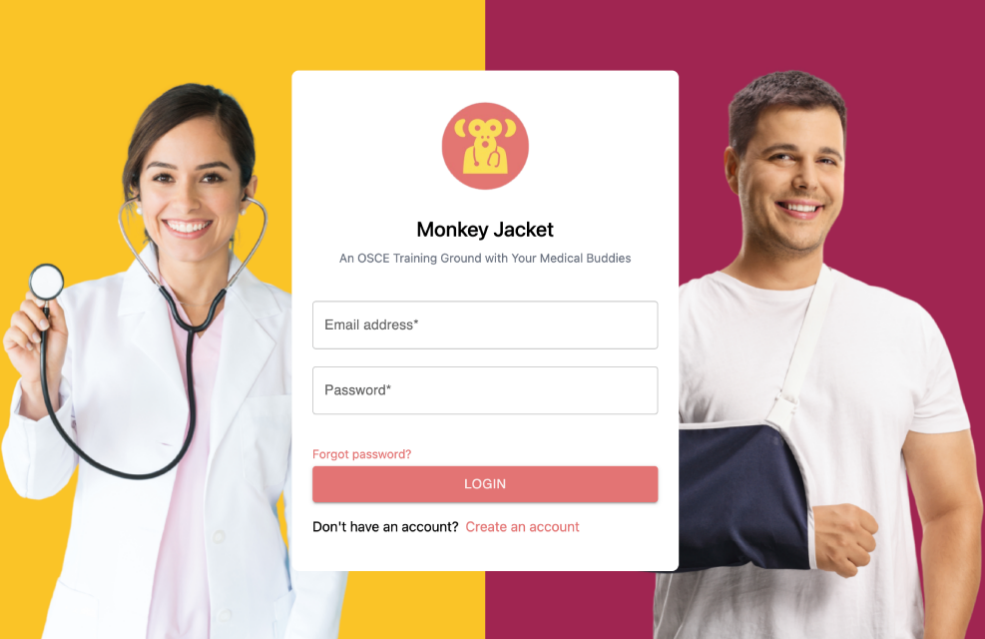
Main log-in screen of the MonkeyJacket platform. OSCE: objective structured clinical examination.

**Figure 2.**
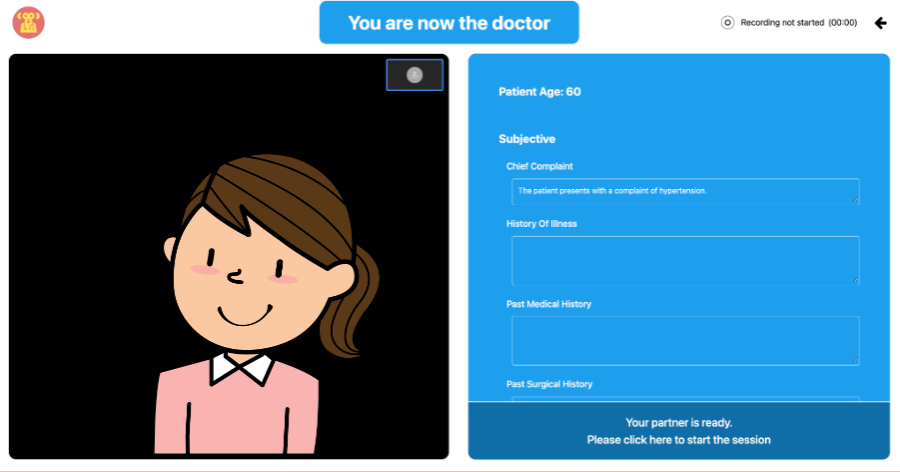
Example screen of the student in the role of the physician. The student physician is able to see the student patient on the left side of the screen and a blank clinical note that may be filled during the encounter.

Upon entry into the platform, students land on a home page in which they are able to enter their email and password credentials (Figure 1). Prior to the start of an OSCE station, the student completing the station as the acting physician receives a brief prompt that introduces the patient’s name, age, and chief complaint (Figure 2).

**Figure 3.**
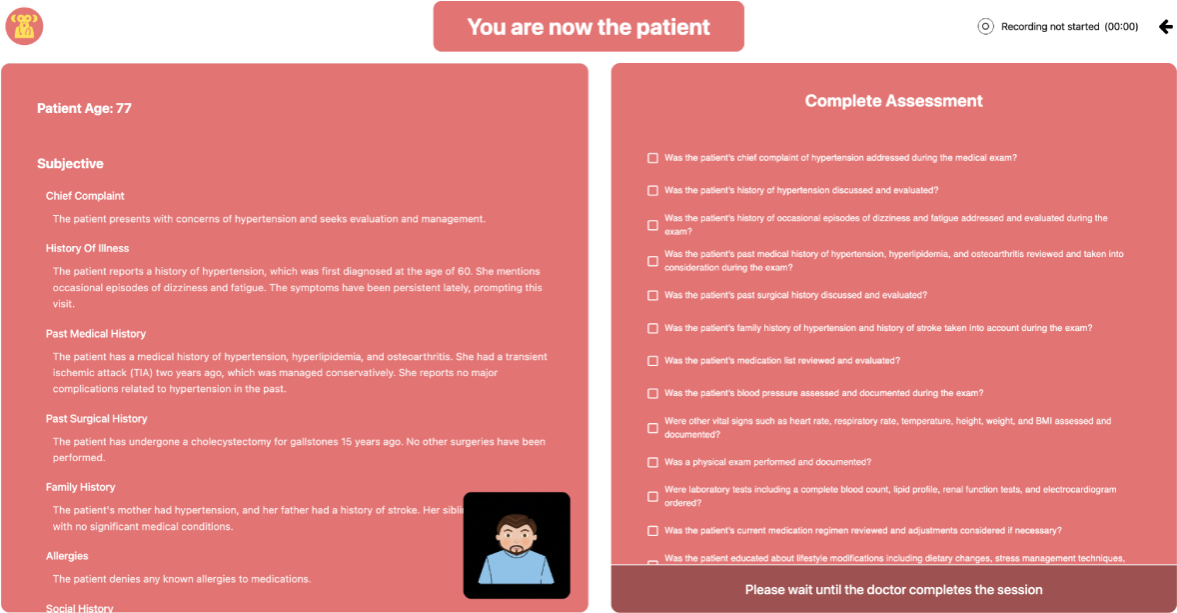
Example of the MonkeyJacket platform screen as seen by the student in the role of the standardized patient. The case details are shown on the left, and the checklist assessment is shown on the right.

Once both students press “Begin station,” the practice OSCE station starts, and the session begins. In this example, student A is practicing their skills as the “physician,” and student B is providing feedback as the “patient.” During this time, student A is only able to see the brief clinical prompt entailing the chief complaint. However, student B is able to view a more extensive patient history, along with behavioral cues, and the feedback checklist for items that student A should inquire about during the patient interview. While student A takes the history, student B is responsible for completing the checklist along with answering clinical questions, which are asked by student A, based on the history provided (Figure 3). At the end of the practice OSCE station, student B is responsible for completing the assessment checklist for student A in order to successfully save and submit the practice session.

### Feedback Checklists

Checklist items can be divided into two categories: (1) generic items and (2) items relevant to the presenting concern. Examples of general checklist items can be found in Textbox 1.

Relevant checklist items are those that are pertinent to the primary presenting concerns of the patient. For example, if the patient presents with shortness of breath, some relevant checklist items could include those listed in (Table 2).

At the end of all assessment checklists, the student is also asked to state the top 2 or 3 differential diagnoses based on the history presented. After stating the differential diagnoses, the student is asked for their top diagnosis. There are also other pertinent clinical questions that the student must answer. Examples of other clinical questions include questions about deciding on the most appropriate imaging modality, other diagnostic tests, and the initial management of the clinical presentation.

**Figure 4.**
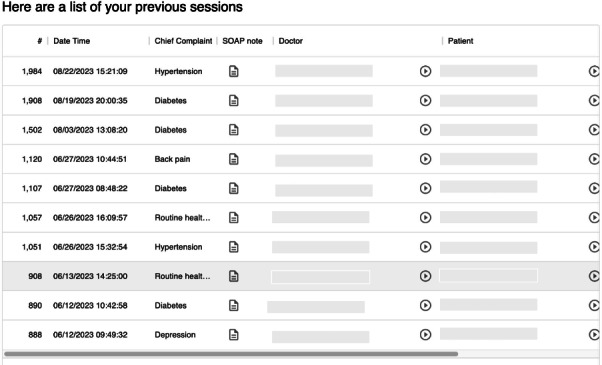
Example of the review screen, through which students may access their scores, their clinical notes, comments from their peers, and an audio file of the encounter. SOAP: Subjective, Objective, Assessment, Plan.

After assessment checklists are completed and submitted on the platform, a percentage score is calculated based on the total number of check marks received. The score is recorded and stored within the MonkeyJacket platform. Students are able to review all personal case attempts that they have completed within the platform. Additionally, audio files are also captured so that students can later review the session and reflect on not just their medical expert knowledge but also the soft skills of communication and rapport building that they must demonstrate (Figure 4).

Textbox 1. **Examples of general objective structured clinical examination checklist items.**

- Introducing self
- Confirming patient’s name and age
- Explaining reason for consult
- Building initial rapport
- Gaining consent
- Asking open-ended questions
- Asking about medications and allergies
- Exploring social history (including cigarettes, alcohol, recreational drugs, diet, occupation, and physical activity)
- Exploring and responding to ideas, concerns, and expectations
- Showing empathy
- Avoiding jargon
- Summarizing issues back to patient
- Global score
- Answering follow-up questions correctly

**Table 2.** Examples of relevant objective structured clinical examination checklist items, with the primary presenting concern being shortness of breath.

Assessment checklist items	Examples of what should be asked about
Asking qualifying questions about presenting symptoms	Onset, duration, site, character, severity, duration, and timeline of pain
Asking about relevant associated symptoms	Coughing, recent calf pain, palpitations, fever, and chest pain
Asking about recent illnesses and past medical history	Heart disease, stroke, diabetes, hypertension, etc
Asking about relevant family history	Heart disease among family members aged younger than 55 y, diabetes, high cholesterol, autoimmune disease, history of atopy, etc

## Discussion

### Overview

The MonkeyJacket application is a novel, innovative, and unique tool for medical students seeking additional practice regarding the development of clinical skills. The overarching goal of the MonkeyJacket application is to fill the gap that exists within medical education—a lack of scalable formative feedback for clinical skill development for learners. The MonkeyJacket application addresses this gap through the focus on peer feedback and the technological features built within the platform. Additionally, the application keeps track of participants’ scores so that individuals may review the trend in and learn from their performance after practice sessions.

The biggest advantage of this platform is the potential for scalability it provides for medical learners. According to Medical Education Statistics 2020, there were 14,967 faculty members and 11,865 medical learners across Canadian medical schools by the beginning of 2020 [15]. On top of the clinical responsibilities of faculty members, they are also responsible for fulfilling teaching and academic requirements. As such, it is not feasible for faculty members to provide additional feedback to learners outside of the designated OSCE preparation time. The MonkeyJacket platform allows students to receive an abundance of feedback from peers, should they wish for additional practice. The scalability of the platform also decreases the administrative load on medical schools, as students would have simple access to additional clinical skills feedback that does not require constant faculty supervision.

Another significant advantage of the MonkeyJacket application is the remote nature of the web-based platform. Traditionally, practice OSCE examinations have been conducted in person, often with a student’s peer or friend. The utilization of the MonkeyJacket application is simple, in that it allows a student to share the link with anyone that has access to a computer and internet connection, thus allowing students to practice regardless of their geographical location. Moreover, medical students would be able to practice with students from other schools, thus promoting interinstitutional learning. A medical student residing in British Columbia could easily practice history-taking skills with a fellow student in Ontario, thus allowing both students to learn from each other and teach each other strategies that they have learned within their respective curricula. It is known that medical education institutions across Canada place emphasis on different areas of focus. For example, it was found that preclerkship pediatric clinical skills training greatly varied across the 17 Canadian medical schools, with 6 schools dedicating less than 7 hours and 8 schools dedicating over 10 hours—a total difference of 30% [16]. The development of a remote-based platform allows medical students to learn from their peers, who may have had more exposure within certain areas when compared to students’ own training, thus enhancing their knowledge.

In addition to the remote nature of the application, it also poses a great advantage in terms of its accessibility with respect to cost. A significant barrier to finding accessible practice resources for medical students is the cost associated with purchasing resources. It was found that, on average, osteopathic medical students spend US $4129 on resources exclusively in preparation for their board examinations [17]. Although this finding is specific to medical students in the United States, where there are different board examinations, Canadian medical students are not exempt from such costs. Canadian medical graduates, on average, finish medical school with CAD $164,688 (US $846,612 as of the time of writing) of debt, including education-related and non–education-related expenses [18]. Although numerous companies offer preparation courses, these can vary in cost from a few hundred dollars to several thousands of dollars. Thus, costs associated with expensive preparation courses and resources can be a significant barrier for students seeking resources. The MonkeyJacket platform is completely open access and free of charge. For medical students looking to gain extra practice, the MonkeyJacket platform provides a simple and accessible option, with multiple opportunities for peer evaluation and progress tracking.

### Limitations

To ensure that the MonkeyJacket web application was serving its intended population, relevant feedback from medical students and residents was taken into consideration when developing the functions and design of the web application. Nonetheless, there were some limitations to this study.

One limitation of this study is the sample size of students included in the feedback process. In this study, there were 5 medical students and 1 medical resident involved throughout the testing process. At the time of writing, the 6 participants have completed over 200 practice case scenarios via the MonkeyJacket platform. Future studies should include a larger sample size of participants in order to obtain more diverse feedback regarding the functionality and usability of the application.

Another limitation of this study is that all participants were from either Western University or McMaster University. This application originated from researchers based in Western University, and thus all students were recruited from the same institution for ease of organization and planning. Although this was advantageous, as the knowledge and OSCE skills were standardized among study participants, this can also reflect a lack of diversity in perspectives with respect to OSCE skills.

Lastly, traditional OSCE examinations are extensive, in that they also evaluate a candidate’s ability to perform relevant physical examination and procedural skills in response to a primary patient concern. Given the web-based nature of the MonkeyJacket platform, it was not possible to integrate such assessments. However, one way to assess a candidate’s knowledge regarding relevant physical examination skills is to add it to the checklist and ensure that the candidate knows the rationale for why certain physical examination components would be used.

### Future Directions

In the future, the MonkeyJacket application will be preparing for extensive nationwide deployment across Canadian medical institutions. Through partnership with major Canadian medical student groups, the application will be disseminated for widespread use. This will allow us to collect a vast amount of quality improvement feedback. Ideally, we will be able to test if the use of the application leads to improved medical examination scores.

At the time of writing, the cases included within the platform are tailored toward scenarios that can help medical learners, who will become competent resident physicians, develop clinical skills. The expansion of the application in the future can include more specialized cases for specific residency subspecialties. In addition, MonkeyJacket is useful not only for Canadian medical students but also for medical trainees globally, as clinical skills examinations are part of many international medical education programs. This can be explored in the future, once the application is successfully deployed in Canada.

### Conclusions

The MonkeyJacket OSCE tool is a comprehensive and accessible learning resource for medical learners. This innovative tool offers medical learners a solution that addresses the lack of practice tools and formative feedback within the realm of clinical skill development. As medical students proceed through their training, OSCEs remain an integral component of assessments ensuring that learners are demonstrating required competencies for safely practicing medicine upon graduation. The development of comprehensive and accessible OSCE practice tools with built-in evaluations eases the stress associated with preparation for clinical examinations and promotes a more competent medical workforce, with the latter benefiting the most important stakeholders in medicine—the patients.

## Abbreviations

CanMED: communicator, collaborator, leader, health advocate, scholar, professional, and medical expert
e-OSCE: electronic objective structured clinical examination
MCC: Medical Council of Canada
OSCE: objective structured clinical examination
